# Development of a novel heterologous gene expression system using earthworms

**DOI:** 10.1038/s41598-021-87641-w

**Published:** 2021-04-14

**Authors:** Shin-ichi Akazawa, Yu Machida, Aya Takeuchi, Yuka Wakatsuki, Naoki Kanda, Norito Kashima, Hayato Murayama

**Affiliations:** Department of Materials Engineering, National Institute of Technology, Nagaoka College, 888 Nishikatakai, Nagaoka, Niigata 940-8532 Japan

**Keywords:** Genetic engineering, Genetic engineering, Genetic techniques, Gene expression analysis, Biotechnology

## Abstract

In recent years, animals and plants have received increasing attention as potential next-generation protein production systems, especially for biopharmaceuticals and animal proteins. The aim of the present study was to develop the earthworms *Eisenia fetida* Waki and *Eisenia andrei* Sagami as next-generation animal protein production hosts. These earthworms have been approved as model animals for acute toxicity tests by the Organization for Economic Co-operation and Development, and they have post-translational modification systems. However, so far, none of the studies have used earthworm transfection techniques. Thus, we developed a transfection method for *E. fetida* and *E. andrei* using microinjection and electroporation systems. The maximum survival rates and transfection efficiencies were 79.2% and 29.2% for *E. fetida*, and 95.8% and 50.0% for *E. andrei*, respectively. Furthermore, human erythropoietin was detected in the transformed earthworm tail fragments using an enzyme-linked immunosorbent assay. These results contribute to the development of a potential earthworm-based novel animal protein production system.

## Introduction

The usefulness of *Escherichia coli* for the production of biopharmaceuticals and animal proteins is limited, owing to the lack of post-translational modification systems in prokaryotes^1^. Hence, various eukaryotic heterologous gene expression systems (hosts), including yeast, other fungi, insects, and animal cells, have been developed. Chinese hamster ovary (CHO) cells have been transfected to produce human erythropoietin (hEPO), which is used as a therapeutic agent against renal diseases^2^. However, the manipulation of these cells is complicated, and the production costs are very high due to the need for CO_2_ incubators. Furthermore, although the demand for antibodies and vaccines is increasing^3^, the currently available production systems are suboptimal. Therefore, animals and plants are receiving increasing attention as potential next-generation protein production systems, called ‘humanoid animals/plants’, in an attempt to solve these problems^3–7^. Humanoid animal/plant production systems are expected to contribute to the production of orphan drugs (drugs for rare diseases), antibiotics, and vaccines^7,8^, which are particularly challenging in terms of profitability; thus, these new systems can reduce the initial investment needed for industrial plant construction^3,9^.

The aim of this study was to develop the earthworms *Eisenia fetida* Waki and *E. andrei* Sagami (closely related species with similar characteristics) as next-generation animal protein production hosts. These earthworms can express human proteins because they possess the necessary post-translational modification systems^10,11^. *E. fetida* and *E. andrei* have been approved as model animals for acute toxicity tests, such as for heavy metal soil pollutants, by the Organization for Economic Co-operation and Development (OECD), as they can be easily obtained and reared^12^ and they express various digestive enzymes, including amylase and cellulase^13^. Therefore, earthworms have been studied worldwide to investigate environmental pollutants and biomass utilisation. Furthermore, recombinant proteins can be harvested as secretory proteins in coelomic fluid^14^. *E. fetida* is an edible earthworm, and we have already developed it as a dietary supplement^15–17^. Therefore, humans can ingest earthworms containing recombinant proteins as a medicinal product in the future.

Kim et al. developed a method for generating transgenic *Perionyx excavatus* earthworms, which is protected by a patent^18^. Despite the widespread interest in *Eisenia* earthworms, none of the studies have reported methods to genetically engineer them. In this study, we developed an earthworm transformation protocol and assessed its potential to produce hEPO.

## Results

### Development of a method for earthworm tail transfection

**Figure 1:**
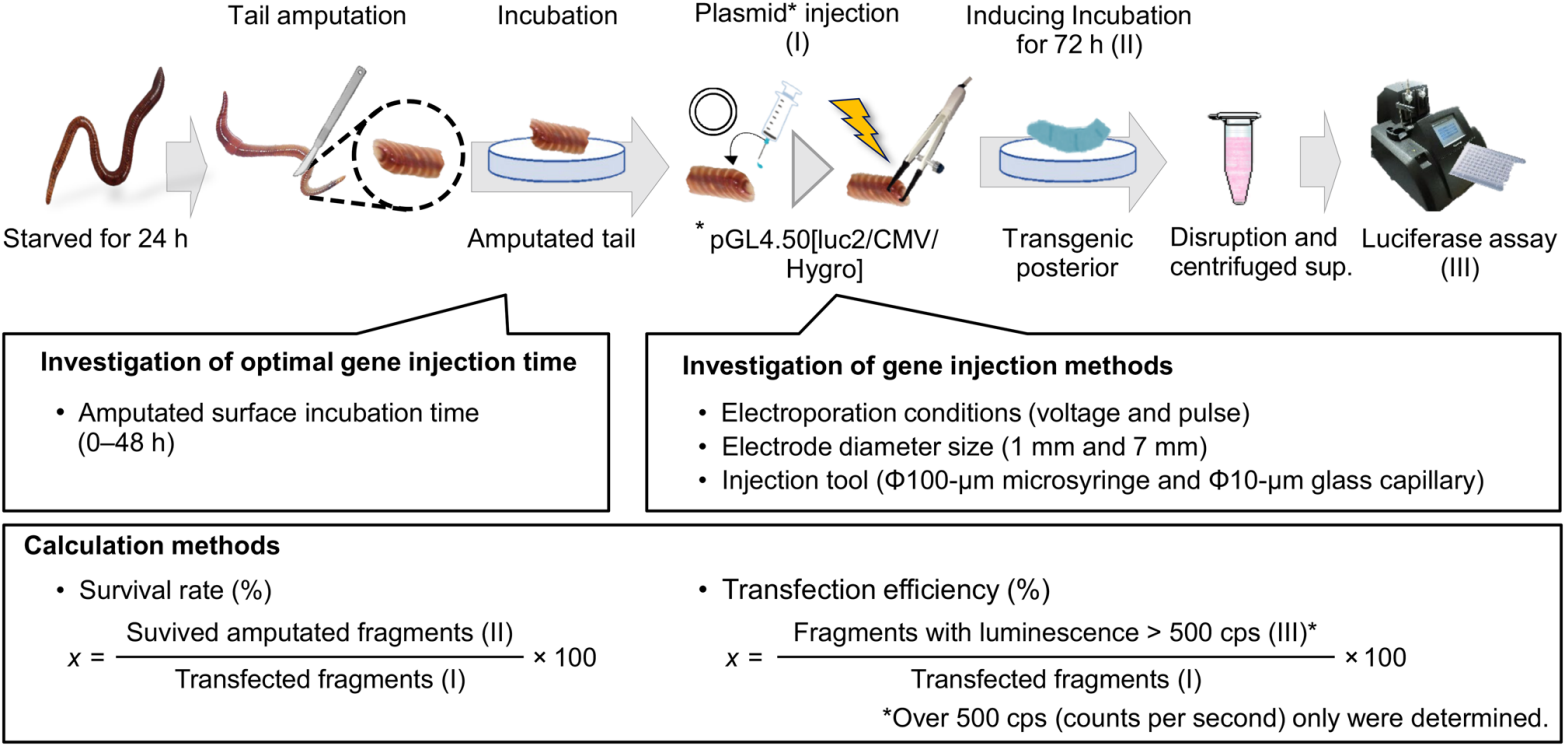
Overview of the earthworm tail transfection method. For easy manipulation, the transfection protocol was developed using an amputated tail fragment. A luciferase expression plasmid was injected into the amputated tail surface, and transfection success was assessed through luciferase luminescence measurements.

**Figure 2:**
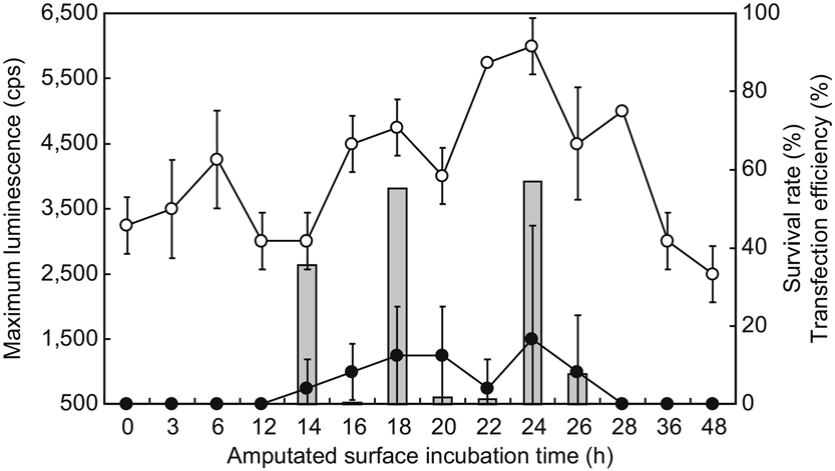
Effect of amputated tail surface incubation time on survival rate and transfection efficiency of *E. fetida*. The electroporation conditions were 35 V and 3 pulses/s at 1 s intervals for 60 ms. All experiments were performed using three groups of eight randomly selected mature earthworms. Open circles, survival rate; closed circles, transfection efficiency; bar graphs, maximum luminescence intensity; cps, counts per second.

**Figure 3:**
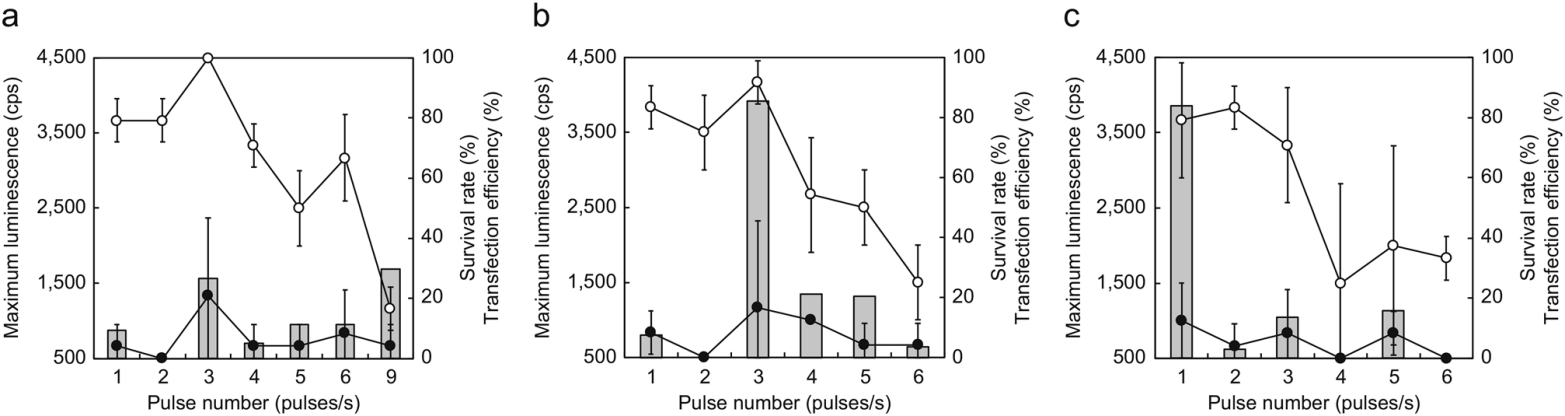
(**a**–**c**) Effect of voltage and pulse number on the survival rate and transfection efficiency of *E. fetida*. The electroporation conditions included a voltage of 28 V (**a**), 35 V (**b**), and 42 V (**c**), and 1–9 pulses/s at 1 s intervals for a duration of 60 ms. All experiments were performed using three groups of eight randomly selected mature earthworms. Open circles, survival rate; closed circles, transfection efficiency; bar graphs, maximum luminescence intensity.

A transfection method was developed, in which *luc2* was incorporated into the amputated tail fragments of *E. fetida* and *E. andrei* (see scheme in Fig. 1). The cut surface incubation time correlated with transfection efficiency, which was also reported by Kim et al.^18^. Closure of the wounded cut surface depends on time (see Supplementary Fig. S1 online). Therefore, we attempted to determine the optimal gene injection time using amputated tail fragments of *E. fetida*. When the transfection conditions were not suitable or the wound could not be closed, the fragment deliquesced, as observed in case of death (see Supplementary Fig. S2 online). Thus, the term ‘survival rate’ has been used in this study. First, we investigated the optimal transfection time. At 18 and 24 h, although there were no statistically significant differences in the transfection rate, the survival rate was significantly different (p < 0.05). In addition, maximum luminescence was observed at 24 h, indicating that the optimal transfection time was 24 h (Fig. 2). Next, we determined the optimal electroporation conditions using *E. fetida* tail fragments, including incubation for 24 h, via experiments at 28 V (Fig. 3a), 35 V (Fig. 3b), and 42 V (Fig. 3c) using 1–9 pulses/s at 1 s intervals for 60 ms and an electrode of 1 mm diameter. We found no significant differences in the transfection rate (*p* > 0.05). Thus, we determined the optimal conditions based on the survival rate and maximum luminescence to be 35 V at 3 pulses/s; the corresponding survival rate and transformation efficiencies were 91.7% ± 7.2% and 16.7% ± 7.2%, respectively (Fig. 3b). Transfection of *E. andrei* under the same conditions was also successful, resulting in a survival rate of 62.5% ± 0% and transfection rate of 12.5% ± 0%.

### Effect of electrode diameter size on transfection efficiency and viability

**Figure 4:**
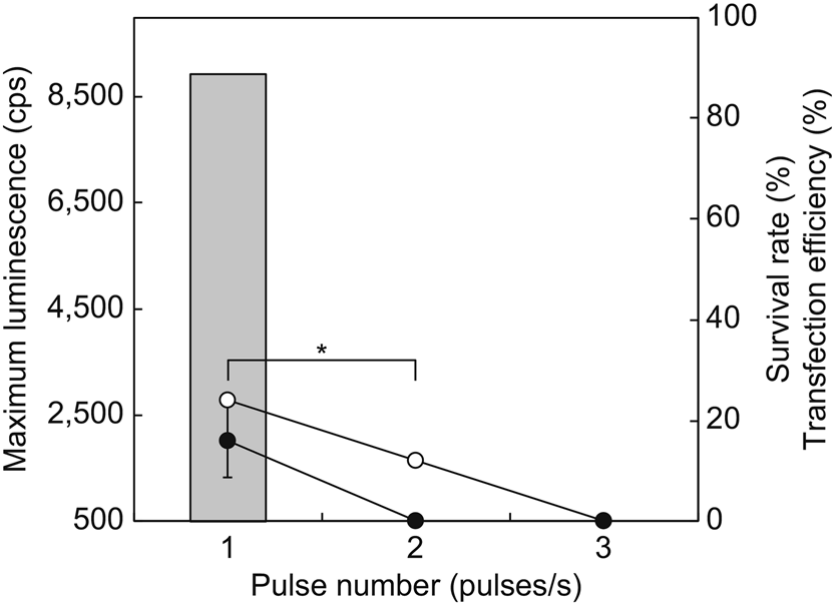
Effect of pulse number using a 7 mm diameter electrode on survival rate and transfection efficiency of *E. fetida*. The electroporation conditions were 35 V, 0 s intervals, and a 60 ms duration. All experiments were performed using three groups of eight randomly selected mature earthworms. Open circles, survival rate; closed circles, transfection efficiency; bar graphs, maximum luminescence intensity. *p < 0.05.

**Figure 5:**
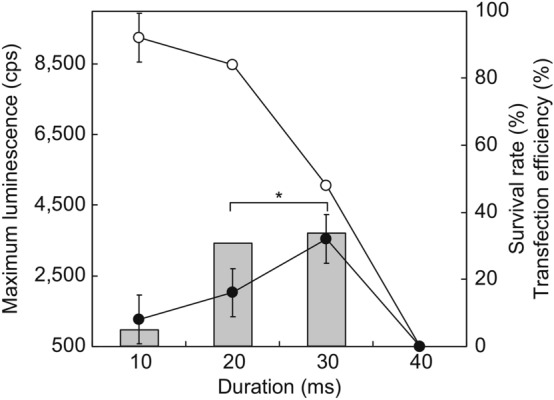
Effect of pulse duration using a 7 mm diameter electrode on survival rate and transfection efficiency of *E. fetida*. The electroporation conditions were 35 V and 1 pulse/s at 0 s intervals for each duration. All experiments were performed using three groups of eight randomly selected mature earthworms. Open circles, survival rate; closed circles, transfection efficiency; bar graphs, maximum luminescence intensity. *p < 0.05.

To improve the transfection efficiency, the electrode diameter was enlarged from 1 to 7 mm. Since none of the tail fragments of *E. fetida* survived upon using the 7 mm electrode under the predetermined optimal electroporation conditions (35 V, 3 pulses/s, 60 ms duration, and 1 s interval), our first experiment was performed at 1–3 pulses/s and 0 s intervals for 60 ms (Fig. 4). Consequently, at 1 pulse/s, the maximum survival rate and transfection efficiencies were 25.0% ± 0% and 16.7% ± 7.2%, respectively (Fig. 4). Furthermore, we assessed the duration of electroporation conditions from 10–40 ms at 1 pulse/s and a 0 s duration (Fig. 5). The maximum survival rate decreased to 50.0% ± 0% (compared to when we used a 1 mm electrode) in the 30 ms duration. The maximum transfection rate was 33.3% ± 7.2% at the 30 ms duration; however, there were no significant differences (*p* = 0.10, 16.7% ± 7.2%) compared to the previous optimal conditions (1 mm diameter electrode, 35 V, 3 pulses/s, 1 s intervals for a duration of 60 ms). In addition, the survival rate and transfection efficiency of *E. andrei* under the same conditions were not significantly different (37.5% ± 0% and 16.7% ± 7.2%, respectively; *p* > 0.05).

### Effect of the gene injection tool on transfection efficiency and viability

The survival rate decreased upon the use of needles with a large diameter. One of the reasons was physical damage to the earthworms, affecting their viability. Thus, the needle was changed from a 100 μm microsyringe (Hamilton Co., Reno, NV, USA) to a 10 µm glass capillary electro-microinjection system (BTX Molecular Delivery Systems, Holliston, MA, USA). Electroporation conditions were the same as mentioned earlier (7-mm diameter electrode, 35 V, 1 pulse/s, 0 s interval, and 30 ms duration).

The survival rates of *E. fetida* (79.2% ± 7.2%) and *E. andrei* (95.8% ± 7.2%) obtained using the 10 µm glass capillary electro-microinjection system were significantly different from those obtained using the 100 μm microsyringe method (*p* < 0.05). In addition, although the transfection efficiency of *E. fetida* (29.2% ± 7.2%) had no significant differences compared with those obtained by the 100 μm microsyringe method (*p* > 0.05), *E. fetida* transfection efficiency (50.0% ± 0%) was significantly different compared with that achieved using the 100 μm microsyringe method (*p* < 0.05) (Table 1).

**Table 1:** Optimal transfection conditions for *E. fetida* and *E. andrei*.

Earthworm	Injection tool	Electrode diameter (mm)	Base medium	
*E. fetida*	ϕ100 μm Microsyringe*	1*	Moist Kimwipe*	
	ϕ10 μm Glass capillary	7	0.6% agar with amp	
*E. andrei*	ϕ10 μm Glass capillary	7	0.6% agar with amp	

	Electroporation conditions			
	Voltage	Pulses/s	Duration (ms)	Interval (s)
*E. fetida*	28*	6*	60*	1*
	35	1	30	0
*E. andrei*	35	1	30	0

	Survival rate (%)	Transfection efficiency (%)		
*E. fetida*	15*	11*		
	79.2 ± 7.2	29.2 ± 7.2		
*E. andrei*	95.8 ± 7.2	50.0 ± 0		

*Initial conditions. Amp, ampicillin. All experiments were performed using three groups of eight randomly selected mature earthworms for each experimental condition, except for initial conditions (average score of 20 earthworms).

### Development of a transfection method for earthworms

**Figure 6:**
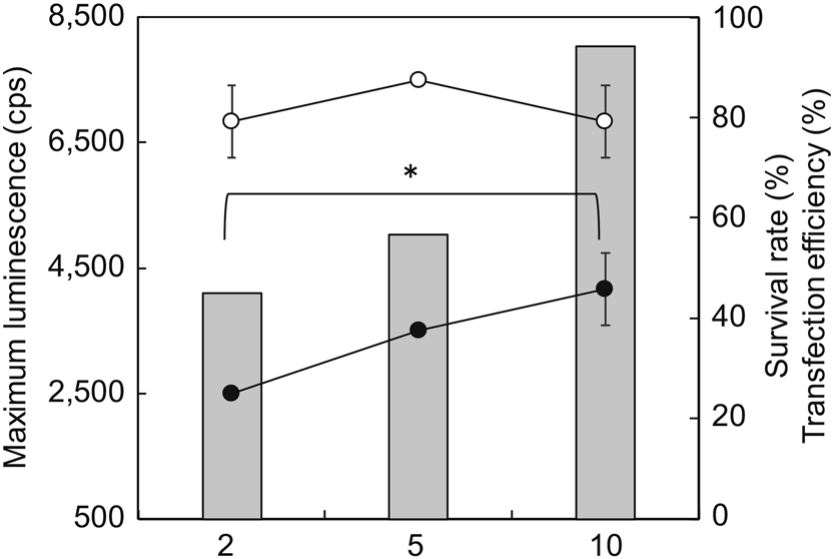
Effect of plasmid amount on the survival rate and transfection efficiency of *E. andrei*. The electroporation conditions were as follows: electrode diameter, 7 mm; pulse voltage, 35 V; pulse rate, 1 pulse/s at 0 s interval; and duration, 30 ms. Open circles, survival rate; closed circles, transfection efficiency; bar graphs, maximum luminescence intensity. *p < 0.05.

We attempted to apply the tail fragment transfection method to the amputated anterior fragments of *E. andrei* using a similar approach. However, the transfection rate was not high (25.0% ± 0%) using the amputated anterior fragments of *E. andrei* and 2 μg of the plasmid. Therefore, the amount of vector was increased from 2 μg to 5 and 10 μg (Fig. 6). As a result, each survival rate was almost the same (around 80%). While the transfection rates with 5 μg and 10 μg of vector did differ significantly, significant differences were observed with 2 μg (25.0% ± 0%) and 10 μg (45.8% ± 7.2%) of the vector. Although further studies are required to determine the optimal DNA amount for transfection, it is evident that the transfection efficiency depends on the amount of DNA.

### Detection of luciferase (*luc2*) transgene by PCR

To verify the incorporation of the transgene into the earthworm genome, we extracted DNA from transfected *E. fetida* tail fragments and from the *E. andrei* anterior fragment of the transfected amputated surface after 1 week of incubation. Although we did not detect a gene fragment corresponding to the estimated size of *luc2* (1,593 bp) in the genome of the untransfected tail and anterior fragments (see Supplementary Fig. S3 lane 3 and 7 online), a *luc2* fragment of the expected size was detected in both transfected samples (see Supplementary Fig. S3 lane 4 and 6 online). *luc2* fragments of the expected size were identified as *luc2* upon sequence analysis.

### Detection of recombinant hEPO from *E. andrei* via enzyme-linked immunosorbent assay (ELISA)

We investigated recombinant hEPO production using an EPO ELISA kit (Roche Diagnostics) following the manufacturer’s instructions. The ability of transfected *E. andrei* tail fragments to produce hEPO was assessed via ELISA and a calibration curve, revealing that a minimal amount (10.1 pg ± 7.3 pg/tail fragment) of hEPO was produced. However, hEPO was not detected in non-transfected *E. andrei* (negative control) (see Supplementary Fig. S4 online).

## Discussion

Recently, Kim et al*.*^18^ reported a transformation method using the earthworm *Perionyx excavatus*, which has gonad-regeneration capability. They injected a gene into the earthworm within 24 h of gonad amputation and it was subsequently incorporated into fibroblast cells during regeneration^18^. However, detailed methods are not available because the method is protected by a patent; hence, the details have not been published. Furthermore, the habitats and cultivation areas of this earthworm are limited, with no farms cultivating this earthworm in Japan. Thus, we constructed a convenient transfection system using commonly used earthworms, *E. fetida* and *E. andrei*, which are used as a research model worldwide because the OECD has designated them as model earthworms for toxicity tests^12^. Kim et al*.*^18^ reported that their transfection method involved only cutting and microsyringe injection. However, we could not successfully transfect *E. fetida* and *E. andrei* using this method. Therefore, we developed a novel transfection method involving electroporation^14^.

Transfection efficiency was associated with the incubation duration of the amputated surface; earthworms gradually repair their amputated surface (see Supplementary Fig. S1 online). This finding is also supported by the findings of Kim et al*.*^18^. If normal cells receive a stimulus such as amputation, the cells dedifferentiate into fibroblast cells, which are necessary for body regeneration^19^. At an incubation duration of > 24 h, fibroblasts differentiated into other cells such as epidermal cells, while incubation durations of < 24 h resulted in a lack of differentiation. Thus, the incorporated plasmid could not proliferate well, resulting in low transfection efficiency and production.

It was somewhat difficult to establish the optimal transfection conditions based on numerous variables (see summary in Table 1). This was especially due to the significant differences in the survival rate in each experimental condition, even though the transfection rate did not vary significantly in each experimental condition. Since the transgenic tail fragments die easily (see Supplementary Fig. S1 and S2 online), the standard deviations were, at times, large (Figs. 2 and 3). Moreover, although we chose the same types of earthworms with similar body size and morphology (mature earthworms that had clitella), we considered that there may be individual differences among earthworms. However, we observed some striking trends in the data from the whole experimental conditions. The larger (7 mm vs. 1 mm diameter) electrode improved the transfection efficiency because it could easily hold the body of the earthworm, including the gene-injected surface. Thus, the electric current could travel through the whole gene-injected segment; the body diameter of the earthworms was approximately 3–5 mm. Furthermore, the needle with the smaller diameter (10 μm glass capillary vs. 100 μm microsyringe) resulted in a higher survival rate and transfection efficiency compared to that obtained upon using the large diameter needle. Upon using a 1 mm diameter electrode and microsyringe for the transfection of *E. andrei*, the survival and transfection rates were 62.5% ± 0% and 12.5% ± 0%, respectively. However, upon using the 7-mm diameter electrode and glass capillary for transfection, the survival and transfection rates were 95.8% ± 7.2% and 50.0% ± 0%, respectively (Table 1). These data show a statistically significant difference (*p* < 0.05), indicating that our experimental settings were appropriate. These values are expected to further improve with additional studies and optimisation of conditions.

hEPO was produced in *E. andrei*, although the amount was very low, probably due to the ineffective cytomegalovirus (CMV) promoter in earthworm gene expression systems. To improve productivity, it would be necessary to include a strong promoter derived from earthworms. We have not confirmed hEPO activity; however, when recombinant hEPO produced by *P. excavatus* was injected into mice, the amount of haemoglobin increased significantly^18^. Furthermore, *E. fetida* has an N-type sugar chain modification mechanism^10,11^. Since the active form of hEPO is formed by the binding of three N-type sugar chains, this earthworm may produce high amounts of active hEPO.

Generally, the development of transgenic animal systems is associated with issues including prohibitive costs and ethical implications^3^. However, earthworms can be reared at a low cost because of their small body (body length is about 5–8 cm) and they feed on waste vegetables. Furthermore, no ethical regulations restrict the use of earthworms. Therefore, the transfection methods developed herein would be potentially useful to a lot of researchers across scientific disciplines. Furthermore, this method provides a new host system for the gene expression analysis of higher organisms. The method developed herein involves a transient expression system, and the recombinant protein productivity is low. However, we have attempted to construct a stable expression system, using eggs and earthworm-predicted promoter regions. Therefore, we expect advancements in the transfection and recombinant protein production systems. These results may potentially contribute to the development of novel heterologous gene expression systems for producing biopharmaceuticals.

## Methods

### Materials

The earthworm *E. fetida* Waki was kindly provided by Waki Pharmaceutical (Nara, Japan), and *E. andrei* Sagami was purchased from Sagami Joka Service Inc. Ltd. (Kanagawa, Japan). Experimental earthworms were mature individuals with clitella. The plasmid pGL4.50 [luc2/CMV/Hygro], encoding the luciferase reporter gene *luc2* (*Photinus pyralis*) and CMV promoter; luciferase assay reagent; and luciferase cell culture lysis reagent were purchased from Promega Co. (Tokyo, Japan). The pRC210775 [hEPO/CMV] plasmid, which consists of the pCMV6-Entry vector and encodes the CMV promoter and human erythropoietin (Myc-DDK-tagged-EPO), was purchased from Origene Technologies Inc. (Rockville, MD, USA). The EPO ELISA kit was purchased from Roche Diagnostics (Indianapolis, IN, USA).

### Transfection of *E. fetida* and *E. andrei*

All experiments were performed using three groups of eight randomly selected mature earthworms in each experimental condition. *E. fetida* or *E. andrei* were incubated for 24 h without food at 20 °C and 60% humidity in a growth chamber (Nippon Medical & Chemical Instruments Co., Ltd, Osaka, Japan). After starvation, tail fragments (1 cm) were amputated and then inoculated on 0.6% agar plates containing 50 μg/mL ampicillin (amp). The plates were placed in an 800 mL plastic container with a moist paper towel and incubated for 3–48 h in a growth chamber (20 °C, 60% humidity). Thereafter, incubated tail fragments were placed in 3% ethanol at 4 °C for 10 min. The luciferase expression plasmid pGL4.50 [luc2/CMV/Hygro] (2 μg) was injected into the amputated tail surface fragment using a 100 μm diameter microsyringe (Hamilton Co., Reno, NV, USA). Electroporation was performed using a Gemini twin wave electroporator (BTX Molecular Delivery Systems, Holliston, MA, USA) and platinum tweezertrode (1 mm diameter) under the following conditions: voltage of 28, 35, or 42 V; 1–9 pulses/s at 1 s intervals; and duration of 60 ms. The 2 μg plasmid-injected tail fragments were then inoculated on 0.6% agar plates with 50 μg/mL amp. The plates were placed in the plastic container with a moist paper towel, and the container was incubated for 72 h in a growth chamber (20 °C, 60% humidity). Subsequently, the fragments were washed with phosphate-buffered saline and added to 240 mL sterile water in a 2 mL screw cap tube containing glass beads. The fragments were crushed at 4,200 rpm for 20 s using a mini bead-beater (Biospec Products Inc., Bartlesville, OK, USA). Luciferase cell culture lysis reagent was added and the sample was incubated at 22 °C for 25 min. After centrifugation (19,000 × *g*, 4 °C for 5 min), the supernatant was assayed for luciferase activity.

### Luciferase assay and determination of the survival rate and transfection efficiency

The luciferase assay was performed using a luciferase assay reagent (Promega), and the luciferase luminescence intensity was detected as counts per second (cps) using the GloMax-Multi Detection System (Promega) in accordance with the manufacturer’s instructions. The survival rate was determined by dividing the number of samples that survived gene injection by the total number of gene-injected samples (Fig. 1). The transfection efficiency was determined by dividing the number of samples with positive luciferase activity (fragments with luminescence > 500 cps) by the total number of gene-injected samples (Fig. 1).

### Assessment of the effect of electrode diameter and gene injection tool on transfection efficiency and viability

Two electrodes with diameters of 1 mm and 7 mm were tested in electroporation experiments. Furthermore, for gene injection, a 100 μm microsyringe was compared to a 10 μm glass capillary with an electric microinjection system (BEX Co., Tokyo, Japan). Other conditions were the same as those described before.

### Development of a transfection method for earthworms using *E. andrei*

Plasmid pGL4.50 [*luc2*/CMV/Hygro] (2–10 μg) was injected into the amputated anterior fragment 2 cm from the tail surface using a 100 μm diameter microsyringe. Electroporation was performed using a Gemini twin wave electroporator and platinum tweezertrode (7 mm diameter) under the following conditions: 35 V, 1 pulse/s for a duration of 30 ms at an interval of 0 s. Other conditions, including incubation and luciferase activity measurements were as described previously (Sections ‘Transfection of *E. fetida* and *E. andrei*’ and ‘Luciferase assay and determination of the survival rate and transfection efficiency’).

### Detection of the luciferase (*luc2*) transgene via PCR

#### Preparation of genomic DNA

The transfected earthworm fragments (72 h incubated tail or 1 week cultivated anterior section) were separately washed with MilliQ-treated water and ground in a mortar with liquid N_2_. Genomic DNA was extracted following the ISOGENOME procedure (Nippon Gene Co., Ltd., Tokyo, Japan) and further purified through phenol–chloroform extraction and ethanol precipitation. The precipitate was dissolved in MilliQ-treated water.

#### Cloning of luc2

The *luc2* was amplified by PCR using template genomic DNA and a *luc2* 1–24 bp 5′ and *luc2* 1574–1593 bp 3′ primer set (5′-ATGGAAGATGCCAAAAACATTAAG-3′ and 5′-GTCCAACTTGCCGGTCAGTC-3′, respectively). PCR conditions were as follows: 98 °C for 1 min, 98 °C for 10 s, 55 °C for 15 s, 72 °C for 2 min (35 cycles), and 72 °C for 3 min. The purified PCR fragment (1,593 bp) was ligated into a pMD20-T vector using the Mighty TA-cloning Reagent Set for PrimeSTAR (both from TaKaRa Bio Inc., Otsu, Shiga, Japan). *Escherichia coli* strain DH5α competent cells were transformed with the ligated product by heat shock.

### Detection of recombinant hEPO in *E. andrei* via ELISA

The hEPO expression plasmid pRC210775 [hEPO/CMV] (2 μg) was injected into the *E. andrei* amputated tail fragment surface using a 10 μm glass capillary and an electric microinjector. Electroporation was carried out using a Gemini twin wave electroporator and platinum tweezertrode (7 mm diameter) under the following conditions: 35 V, 1 pulse/s for 30 ms with an interval of 0 s. Other procedures, including incubation and sample preparation for the luciferase assay, were performed as described previously. A part of the prepared crude extract was used to detect and determine the concentration of recombinant hEPO. Recombinant hEPO production was analysed using the EPO ELISA kit (Roche Diagnostics) and Corona Multimode Microplate Reader (Corona Electric Co, Ltd., Ibaraki, Japan) in accordance with the manufacturer’s instructions. Anti-hEPO-peroxidase and hEPO human serum were used as a recombinant earthworm hEPO-detecting antibody and positive control, respectively. The non-transfected tail fragments were used as negative control. The concentration of the earthworm-derived recombinant hEPO was determined using a calibration curve prepared using hEPO standard reagents.

### Statistical analysis

All experiments were performed using three groups of eight randomly selected mature earthworms in each experimental condition. Statistical analysis was performed based on these three groups and the standard deviations were calculated. Data are presented as mean ± S.D. Welch’s unequal variances *t*-tests were performed to analyse the data using Microsoft Excel 2016, and results with *p*-values < 0.05 were considered statistically significant.

### Ethical considerations

No ethics regulations restrict the use of earthworms.

## Supplementary Information

Supplementary Information

## Data Availability

Data sharing is not applicable to this article as no datasets were generated or analysed during the current study.
