# Supplementary material for: Development of a novel heterologous gene expression system using earthworms
Source: Sci Rep. 2021 Apr 14;11:8190. doi: 10.1038/s41598-021-87641-w (PMC8046771; doi:10.1038/s41598-021-87641-w)
Supplement: Supplementary file 1 — Supplementary Information [file 41598_2021_87641_MOESM1_ESM.pdf]

SUPPLEMENTARY INFORMATION

Development of a novel heterologous gene expression system using earthworms

Shin-ichi Akazawa*, Yu Machida, Aya Takeuchi, Yuka Wakatsuki, Naoki Kanda, Norito Kashima, and Hayato Murayama

Department of Materials Engineering, National Institute of Technology, Nagaoka College, 888 Nishikatakai, Nagaoka, Niigata 940-8532, Japan

*Correspondence to:
Shin-ichi Akazawa
Department of Materials Engineering, National Institute of Technology
Nagaoka College, 888 Nishikatakai, Nagaoka, Niigata 940-8532, Japan
Tel & Fax: +81-258-34-9255; E-mail: s-akazaw@nagaoka-ct.ac.jp

Key words: Earthworm, *Eisenia fetida*, *Eisenia andrei*, transfection, biomedicine

List of contents

- **Supplementary Figure S1**. Time-course appearance of the amputated tail surface (alive condition).
- **Supplementary Figure S2**. Time-course appearance of the amputated and gene-injected tail (dead condition).
- **Supplementary Figure S3a-b.** Amplified *luc2* fragment from earthworm genomic

DNA of the transfected (a) *E. fetida* tail fragment and (b) *E. andrei* anterior fragment.

- **Supplementary Figure. S4.** Detection of recombinant human erythropoietin (hEPO) from *E. andrei* via enzyme-linked immunosorbent assay (ELISA).

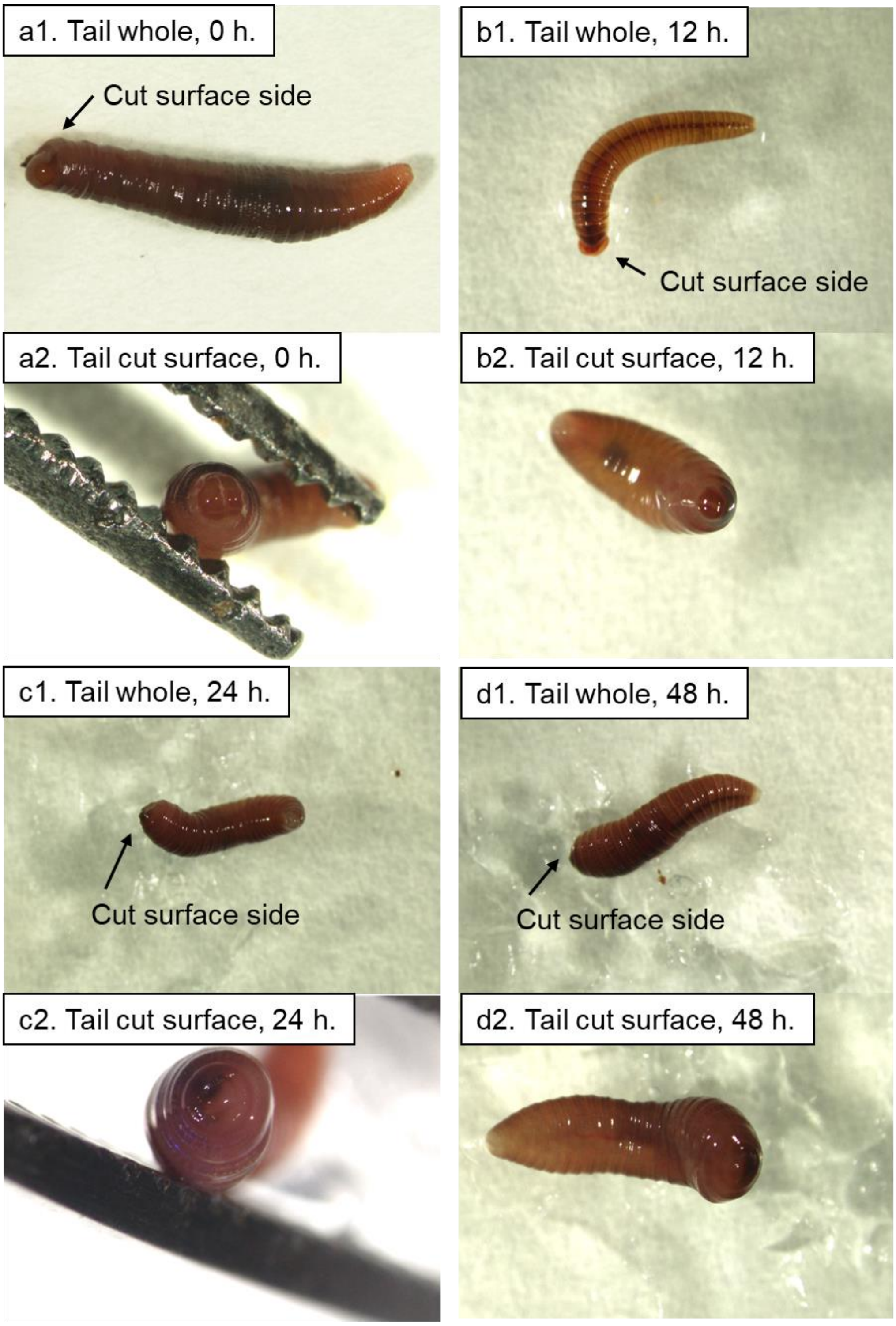

Supplementary Fig. S1. Time-course appearances of the amputated tail surface (alive condition). The amputated tail fragments were inoculated on 0.6% agar plates containing 50 µg/mL ampicillin (amp). The plates were placed in an 800-mL plastic container with a moist paper towel and incubated in a growth chamber (20 °C, 60% humidity). Character a, b, c, d show the results obtained at 0 h, 12 h, 24 h, and 48 h, respectively. Numbers 1 and 2 show the whole picture of gene-injected-tail fragments and the close-up of the cut surfaces, respectively.

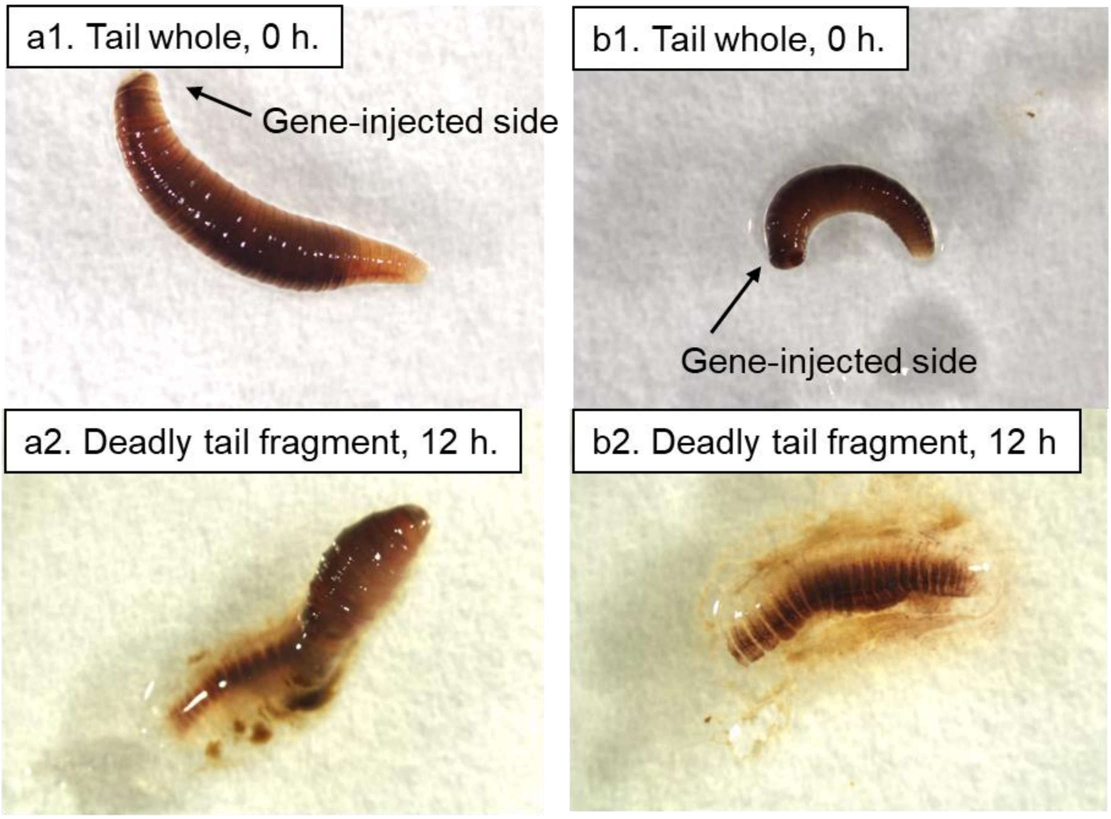

Supplementary Fig. S2. Time-course appearances of the amputated and gene-injected tail fragment (dead condition). The gene-injected tail fragments were inoculated on 0.6% agar plates containing 50 μg/mL ampicillin (amp). The plates were placed in an 800-mL plastic container with a moist paper towel and incubated in a growth chamber (20 °C, 60% humidity). Character a and b show the sample number, respectively. Numbers 1 and 2 show the results obtained at 0 h and 12 h, respectively.

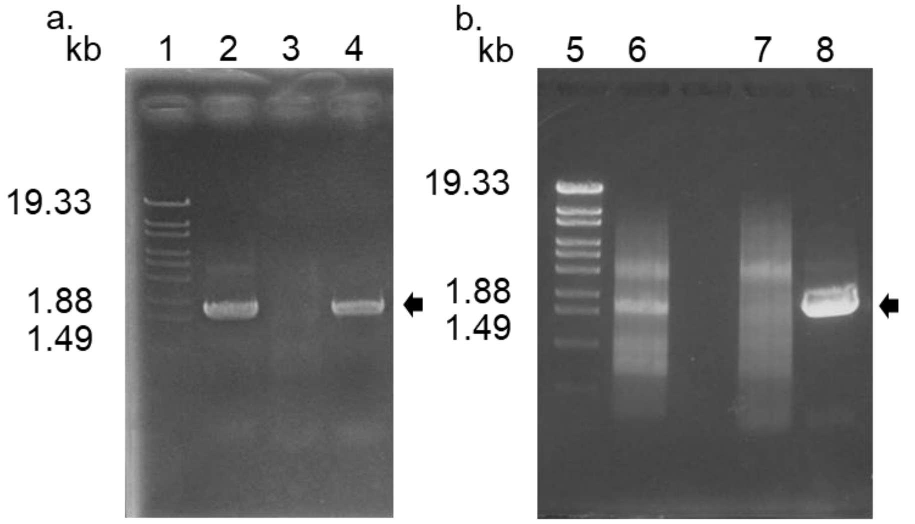

Supplementary Fig. S3a-b. Amplified *luc2* fragment from earthworm genomic DNA of the transfected (a) *E. fetida* tail fragment and (b) *E. andrei* anterior fragment. Lanes 1 and 5, OneSTEP Marker 6 (λ/Sty I digest) (Nippon Gene); lanes 2 and 8, amplified *luc2* fragment from pGL4.50 [*luc2*/CMV/Hygro]; lanes 3 and 7, amplified *luc2* fragment from genomic DNA from untransfected tail and anterior fragments; lane 4, amplified *luc2* fragment from transfected tail fragment; lane 6, amplified *luc2* fragment from transfected anterior fragment.

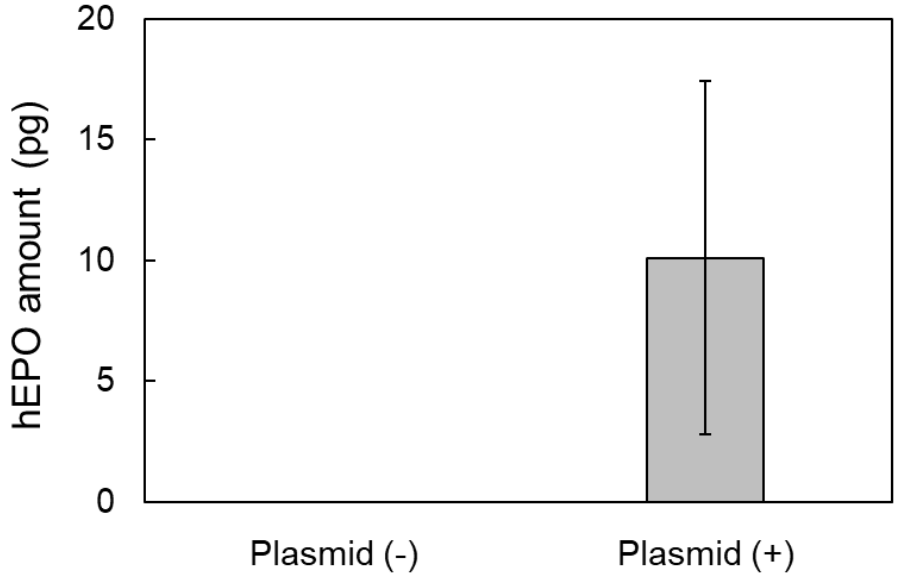

Supplementary Fig. S4. Detection of recombinant human erythropoietin (hEPO) from *E. andrei* via enzyme-linked immunosorbent assay (ELISA). 'Plasmid (+)' indicates that the hEPO expression plasmid pRC210775 [hEPO/CMV] (2 μg) was transfected into the *E. andrei* amputated tail fragment. Recombinant hEPO production was analysed using the EPO ELISA kit. Anti-hEPO-peroxidase and hEPO human serum were used as a recombinant earthworm hEPO-detecting antibody and positive control, respectively. 'Plasmid (-)' indicates the non-transfected tail fragments (negative control).
